# Patient Perceptions and Preferences When Choosing a Surgeon: A Cross-Sectional Study, Qassim Region, Saudi Arabia

**DOI:** 10.7759/cureus.39577

**Published:** 2023-05-27

**Authors:** Reem A Alsalamah, Emad A Aljohani, Renad Aljasser, Jolan S Alsaud, Raghad Alsherbi, Ibrahim Ahmed J Albalawi, Malik M Alreshidi, Fahad H Binshalhoub, Juri A Alhatlani

**Affiliations:** 1 College of Medicine, Qassim University, Qassim, SAU; 2 College of Medicine, Prince Sattam bin Abdulaziz University, Alkharj, SAU; 3 College of Medicine, Taif University, Taif, SAU; 4 Medicine, University of Tabuk, Tabuk, SAU; 5 Collage of Medicine, Taibah University, Medena, SAU; 6 Medicine and Surgery, Imam Muhammad Ibn Saud Islamic University, Riyadh, SAU; 7 Collage of Medicine, Qassim University, Qassim, SAU

**Keywords:** kingdom of saudi arabia (ksa), general surgery, quality improvement, patient decision-making, elective surgery

## Abstract

Background

The patient-physician relationship is changing, and patients are taking more responsibility in their decision-making. Many patients use the Internet as an important source of information regarding their health. Physician-rating websites provide essential information about the quality of care from patients' perspectives. However, choosing the appropriate healthcare provider is still complicated for any patient. Many patients find choosing a surgeon stressful because they cannot change the treating surgeon once the surgery is underway. Understanding a patient's preferences in choosing the right surgeon is essential to forming a patient-surgeon relationship and shaping practice. Nevertheless, little is known about the factors influencing patients' choices for elective surgeries in the Qassim region.

Objectives

This study aims to explore factors and the most common manners patients use to reach their appropriate surgeon in the Qassim Region, Saudi Arabia.

Methods

A cross-sectional study was conducted among target people over 18 years old in Qassim Region, Saudi Arabia, using a snowball sample study from October 2022 to February 2023. The data were collected online using Google Forms using a self-administered, valid Arabic questionnaire distributed to respondents via WhatsApp, Twitter, and Telegram. The questionnaire consists of two sections: participants' sociodemographic status, including age, gender, nationality, residence, occupation, and monthly income; and a section to evaluate factors influencing patient decision-making when choosing a surgeon for elective surgery.

Results

The factors that were significantly associated with elective surgery were: gender of the doctor (adjOR = 1.62, 99% CI: 1.29-2.04); age (adjOR = 1.31, 99% CI: 1.13-1.53); gender of the study patient (adjOR = 1.64, 99% CI: 1.28-2.10); nationality (adjOR = 0.49, 95% CI: 0.26-0.88); and employment (adjOR = 0.89, 95% CI: 0.79-0.99).

Conclusions

The cultural background in the Kingdom of Saudi Arabia plays a significant role in influencing gender in the choice of the surgeon for elective surgery. Recommendations from friends and family members play a less significant role in the choice of the surgeon for elective surgery. Patients in employment and pensioners seem to have a significant preference in the choice of surgeon for elective surgery.

## Introduction

The patient-physician relationship is changing, and patients are taking more responsibility in their decision-making. Many patients use the internet as an important source of information regarding their health. Physician-rating websites provide essential information about the quality of care from patients' perspectives. Physician-rating websites allow patients to write about their experience with a physician or read other patients' evaluations before seeking medical advice [1]. However, choosing the appropriate healthcare provider is still a complicated task for any patient. Many patients find choosing a surgeon stressful because they cannot change the treating surgeon once the surgery is underway [2].

Many variables, including patient coverage, economic concerns, regional systems, and network accessibility, impact healthcare decisions [3]. Patients consider various factors while choosing their physicians and procedures, according to socioeconomic, cultural, and other factors [4]. In recent decades, there has been an increase in studies that focus on the influencing factors patients choose for healthcare providers and health plans [5]. Clinical factors like reputation, professional experience, technical expertise, and the social media presence of a physician are more critical than non-clinical factors like insurance or location [6,7]. Other factors might affect patients' choices, like the type of surgery to be performed, recommendations by family and friends, word-of-mouth, and physician referrals when choosing a surgeon [8,9]. In addition, female patients might prefer surgeons of the same gender for gynecological and breast disorders, which increase from 6% to 14% if a physical examination is suspected [2,10]. According to Huis in’t Veld et al., the majority of female patients do not have a preference for a plastic surgeon's gender. However, patients care more about the surgeon's reputation [11]. Nevertheless, factors like race and religion did not affect most of the patients [12].

On the other hand, these non-clinical criteria might not matter to certain patients thinking about having surgery. In contrast to a family physician involved in the long-term treatment of patients and their families, a surgeon often has a short relationship with a patient. As a result, some patients may base their decision solely on the surgeon's surgical skills [13]. According to Martins et al. [14], 69.6% of respondents felt the most critical factor was the surgeon's reputation. Another study in a nearby country, Lebanon, showed that male surgeons were preferred over female surgeons in most specialties. Abdul Halim et al. [15] add that, in addition, most patients want to participate in decision-making. Individual differences, however, may exist. Knowledge of the elements influencing patients' choices is crucial to including patients in treatment decisions [16]. Therefore, it is advised that the patient and the doctor discuss their options before deciding whether to have surgery. When a patient decides to have surgery, the clinician must share their knowledge and skills with the patient in order to provide the best possible care [17]. Effective, shared decision-making preferences and interaction between the surgeon and the patient reduce pre-surgery anxiety, help the patient understand the surgical procedure better, and enable them to make informed decisions about their healthcare [18,19].

In Saudi Arabia, a conservative society may affect patients' physician choices. It has been noticed that some Saudis prefer male physicians over females, especially in some specialties [2]. Understanding a patient's preferences in choosing the "right" surgeon is essential in forming a patient-surgeon relationship and shaping practice [5,10]. The surgeon's attitudes impact surgery patients' decisions to choose a surgeon more than the doctor's standing, level of training, or online presence [4].

Nevertheless, little is known about the factors influencing patients' choices for elective surgeries in the Qassim region. Therefore, this study aims to explore these factors and the most common methods patients use to reach their appropriate surgeon.

## Materials and methods

Our study is a cross-sectional analytic study to determine the factors influencing patient decision-making when selecting a surgeon for elective surgery. The data were collected using a valid, pretested, structured questionnaire taken from previous studies between October 2022 and February 2023. The study targeted residents over 18 years old in the Qassim region of Saudi Arabia. The questionnaire was distributed online using Google Forms using a self-administered Arabic questionnaire to respondents via WhatsApp, Twitter, and Telegram. A total of 513 respondents were collected for the study. A confidence level of 0.05 and an alpha of 0.5 were used to determine a sample size of 384.

n = (z)^2^ *p (1 - p)/d^2^

where n is the sample size; z is the confidence level (1.96); p is the expected prevalence (50%); d is the absolute error (5%).

The confidentiality of the study participants was assured by not using any patient identifiers on the questionnaire. The self-administered questionnaire had two sections. The first section is the sociodemographic status, including age, gender, nationality, residence, occupation, and monthly income. The second section evaluates factors influencing patient decision-making when choosing a surgeon for elective surgery. All the items that were used to investigate reasons that would prompt an individual to seek a surgeon for elective surgery were measured using a three-point Likert scale: not important (1), important (2), and very important (3). However, to run the logit and probit analyses, they were coded into binary categories: not important (0), while important and very important were coded (1). Both logit and probit regression analysis were used as the main analytical processes using R language version 4.1.2 (RStudio, Boston, MA), and the data manipulation was conducted using the dplyr package.

We obtained ethical approval from Qassim University with reference number 23-24-17. The participants were informed about the purpose of the research, their rights to confidentiality, and the withdrawal procedure from the study. We obtained electronic consent before completing the questionnaire. Data were identified initially and then coded in the database using a Microsoft Excel sheet (Redmond, USA) using a unique identification number. The data were stored on a password-protected laptop with the principal investigator (PI) and corresponding investigator (CI), and all data were maintained confidential. Only the research team had access to the database for analysis purposes. The manuscript included only a summary of the statistics without publication of the raw data, including the identifying information of the participants.

## Results

This section presents the results of the demographics and the multivariate analysis.

Descriptive statistics

Age, gender, nationality, living area, employment, monthly income, and the preferred gender of the doctor were treated as covariates and independent variables. Table 1 shows that age, gender, nationality, employment, monthly income, and the preferred gender of the doctor have a significant association with elective surgery. Thus, these significant covariates were tested further using the probit regression model. More details on specific percentages are provided in Table 1.

**Table 1:** Covariates variables against the dependent variable (elective surgery)

Covariates	Characteristics	No	Yes	Significance value
Age	≤18	31 (6%)	10 (1.9%)	χ² (4) = 52.48, p < 0.001
	19–30	186 (36.3%)	109 (21.2%)	
	31–50	40 (7.8%)	75 (14.6%)	
	51–65	11 (2.1%)	36 (7%)	
	≥65	7 (1.4%)	8 (1.6%)	
Gender	Female	209 (40.7%)	127 (24.8%)	χ² (1) = 28.93, p < 0.001
	Male	66 (12.9%)	111 (21.6%)	
Nationality	Non-Saudi	5 (1%)	18 (3.5%)	χ² (1) = 9.83, p = 0.002
	Saudi	270 (52.6%)	220 (42.9%)	
Living area	Buraydah	113 (22%)	110 (21.4%)	χ² (2) = 1.49, p = 0.475
	Non-Buraydah city	133 (25.9%)	103 (20.1%)	
	Non-Buraydah rural	29 (5.7%)	25 (4.9%)	
Employment	Student	43 (8.4%)	27 (5.3%)	χ² (3) = 74.99, p < 0.001
	Employed	61 (11.9%)	119 (23.2%)	
	Unemployed	6 (1.2%)	26 (5.1%)	
	Retired	165 (32.2%)	66 (12.9%)	
Income (SR)	<5000	173 (33.7%)	74 (14.4%)	χ² (4) = 55.51, p <0.001
	5000–10,000	24 (4.7%)	52 (10.1%)	
	10,000–15,000	29 (5.7%)	50 (9.7%)	
	15,000–20,000	30 (5.8%)	43 (8.4%)	
	>20,000	19 (3.7%)	19 (3.7%)	
Preferred doctors	Female	91 (17.7%)	56 (10.9%)	χ² (2) = 43.05, p < 0.001
	Male	52 (10.1%)	109 (21.2%)	
	No gender preference	132(25.7%)	73 (14.2%)	

The study had 14 independent variables that were tested using chi-statistics to determine which factors were related to the choice of elective surgery (Table 2). The statistically significant predictors with an effect on elective surgery were: recommendations, social media, cost of surgery, gender of the doctor, and hospital reputation. The significant predictors were tested further for their effect using probit regression analysis.

**Table 2:** Independent variables against the dependent variable (elective surgery)

Predictors	Characteristics	No	Yes	Significance value
Recommendations	Not important	19 (3.7%)	41 (8%)	χ² (1) = 13.15, p < 0.001
	Important	256 (49.9%)	197 (82.8%)	
Reputation	Not important	11 (2.1%)	16 (3.1%)	χ² (1) = 1.90, p = 0.168
	Important	264 (51.5%)	222 (43.3%)	
Social media	Not important	171 (33.3%)	125 (24.4%)	χ² (1) = 4.88, p = 0.027
	Important	104 (20.3%)	113 (22%)	
Appointment time	Not important	12 (2.3%)	19 (3.7%)	χ² (1) = 2.94, p = 0.086
	Important	263 (51.3%)	219 (42.7%)	
Cost of surgery	Not important	19 (3.7%)	29 (5.7%)	χ² (1) = 4.19, p = 0.041
	Important	256 (49.9%)	209 (40.7%)	
Accepting insurance	Not important	42 (8.2%)	48 (9.4%)	χ² (1) = 2.11, p = 0.146
	Important	233 (45.4%)	190 (37%)	
Patient’s needs	Not important	4 (0.8%)	8 (1.6%)	χ² (1) = 2.03, p = 0.154
	Important	271 (52.8%)	230 (44.8%)	
Superior skills	Not important	16 (3.1%)	14 (2.7%)	χ² (1) = 0.00, p = 0.975
	Important	259 (50.5%)	224 (43.7%)	
More experienced	Not important	19 (3.7%)	19 (3.7%)	χ² (1) = 0.22, p = 0.643
	Important	256 (49.9%)	219 (42.7%)	
Qualifications	Not important	14 (2.7%)	19 (3.7%)	χ² (1) = 1.77, p = 0.183
	Important	261 (50.9%)	219 (42.7%)	
Gender of doctor	Not important	169 (32.9%)	95 (18.5%)	χ² (1) = 23.70, p < 0.001
	Important	106 (20.7%)	143 (27.9%)	
Personal care and hygiene	Not important	9 (1.8%)	15 (2.9%)	χ² (1) = 2.63, p = 0.105
	Important	266 (51.9%)	223 (43.5%)	
Hospital reputation	Not important	6 (1.2%)	16 (3.1%)	χ² (1) = 6.41, p = 0.011
	Important	269 (52.9%)	222 (43.3%)	
Hospital distance	Not important	80 (15.6%)	78 (15.2%)	χ² (1) = 0.912, p = 0.368
	Important	195 (38%)	160 (31.2%)	

**Figure 1:**
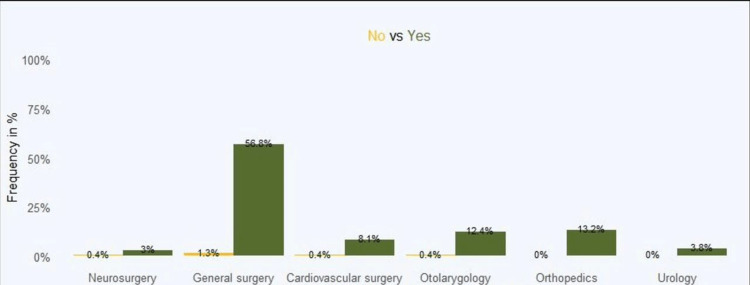
Clinics chosen for elective surgery

**Figure 2:**
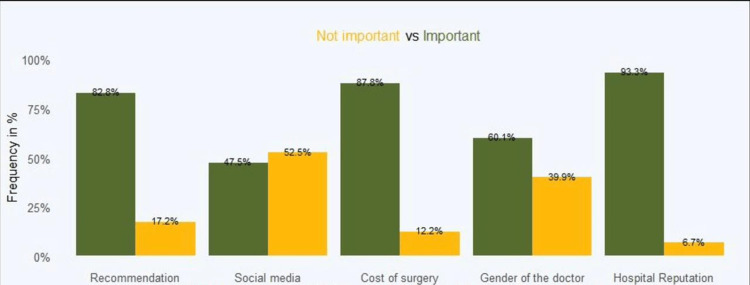
Significant independent factors among those who had undergone elective surgery

Regarding the most attended clinics for elective surgery, general surgery topped the list at 56.8%, followed by orthopaedics (13.2%) and otolaryngology (12.4%). Neurosurgery was the least at 3% (Figure 1). However, it is worth noting that among the significant independent variables, hospital reputation had the highest influence at 93.3%, followed by the cost of surgery (87.9%) and recommendation at 82.8%, while social media was the least influential at 47.5% (Figure 2).

Inferential statistics

The probit regression model was used to test the factors that were statistically significant using chi-statistics: recommendation, social media, cost of surgery, gender of the doctor, and hospital reputation. Recommendation (adjOR = 0.57, 99% CI: 0.40-0.82) and gender of the doctor (adjOR = 1.62, 99% CI: 1.29-2.04) were significantly associated with elective surgery (Table 3).

**Table 3:** Multivariate analysis using the significant independent variables Notes: *p<0.1; **p<0.05; ***p<0.01. Beta=Beta estimates.

Independent variables	Dependent variable: elective surgery	
	Logistic (1) Beta and p-values	Probit (2) Beta and p-values
(1) Recommendation	−0.920; p=0.003***	−0.560; p=0.003***
(2) Social media	0.294; p=0.123	0.180; p=0.126
(3) Cost of surgery	−0.589; p=0.069*	−0.355; p=0.073*
(4) Gender of the doctor	0.780; p=0.00004***	0.483; p=0.00004***
(5) Hospital reputation	−0.682; p=0.181	−0.407; p=0.181
Constant	1.351; p=0.027**	0.804; p=0.027**
Observations (n)	513	513
Log-likelihood	−332.608	−332.702
Akaike Inf. Crit.	677.216	677.404

The probit regression findings that were significantly associated with elective surgery were: the gender of the doctor (adjOR = 1.62, 99% CI: 1.29-2.04); the age of the study participant (adjOR = 1.31, 99% CI: 1.13-1.53); the gender of the study participant (adjOR = 1.64, 99% CI: 1.28-2.10); the nationality of the study participant (adjOR = 0.49, 95% CI: 0.26-0.88); and employment of the study participant (adjOR = 0.89, 95% CI: 0.79-0.99) (Table 4).

**Table 4:** Multivariate analysis using the significant independent and covariate variables Notes *p<0.1; **p<0.05; ***p<0.01. Beta = Beta estimates.

Independent variables	Dependent variable: elective surgery	
	logistic (1) Beta and p-values	Probit (2) Beta and p-values
(1) Recommendation	−0.593; p = 0.065*	−0.363; p = 0.060*
(2) Gender of the doctor	0.787; p = 0.0001***	0.481; p = 0.00005***
(3) Age	0.446; p = 0.001***	0.272; p = 0.0005***
(4) Gender of the participant	0.801; p = 0.0002***	0.493; p = 0.0001***
(5) Nationality	−1.254; p = 0.025**	−0.722; p = 0.025**
(5) Employment	−0.200; p = 0.032**	−0.121; p = 0.034**
Constant	0.649; p = 0.329	0.347; p = 0.368
Observations (n)	513	513
Log-likelihood	−306.940	−306.892
Akaike Inf. Crit.	627.879	627.785

**Figure 3:**
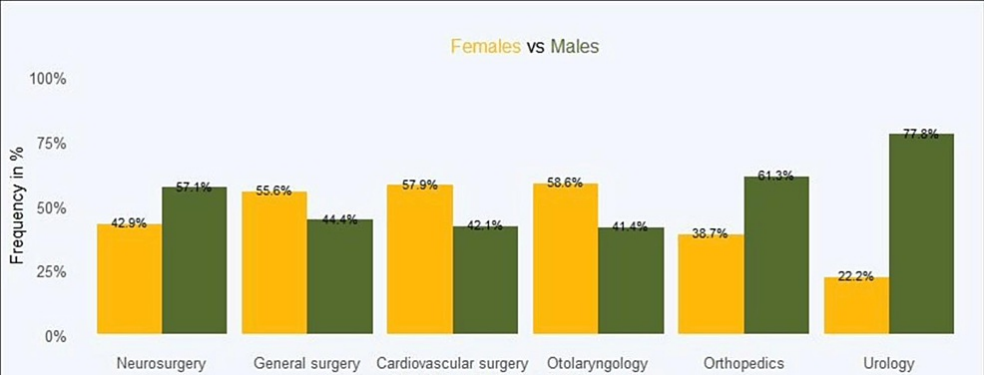
Choice of elective surgery among females and males

Figure 3 shows the positive choice of elective surgery among females and males. It is worth noting that although there were variations in the proportions, the only significant differences were noted in the choice of elective surgery: orthopaedics (χ² (1) = 5.11; p = 0.024) and urology (χ² (1) = 30.91; p < 0.001).

## Discussion

We found a strong positive association between the gender of the doctor and the age and gender of the participant in elective surgery. Both logit and probit regression models showed the gender of the doctor had a significant positive relationship with elective surgery. The preliminary analysis on recommendations and the gender of the doctors in their roles in the choice of elective surgery indicated that only the gender of the doctor had statistically significant findings. The findings are similar to the study by Alkhaldi et al. [20] that showed 49.5% of the participants preferred a surgeon of the same gender when consulting for an elective visit to a surgery clinic, whereas female patients significantly preferred a female surgeon (p < 0.001). However, a study by Hancock et al. [8] showed no preference for male or female surgeons for body procedures.

The study investigated the relationship between gender and elective surgery. Males had no significant relationship with elective surgery. The gender of the doctor had 1.69 times greater odds of influencing the choice of elective surgery among females. However, the recommendation had 2.73 lesser odds of influencing elective surgery among females compared with the gender of the doctor. Our finding shows that, indeed, gender has a significant influence on matters of selecting elective surgery. Studies by Alotaibi et al. [2], Hancock et al. [8], and Hoffman et al. [21] reported that female patients might prefer surgeons of the same gender for gynecological, colorectal, and breast disorders. In the current study, the preference for surgery clinics was statistically significant in orthopedics and urology, with males taking the most considerable proportions in the equations. In the selection of orthopedics, there were more males (61.3%) compared with females (38.7%), and equally, in the choice of urology clinics, there were more males (77.8%) compared with 22.2% females. This finding is similar to that of Alotaibi et al. [2], a study that reported that Saudi Arabia's conservative society may affect patients' choice of physician, with some Saudis preferring male physicians over females, especially in some specialties.

The study examined the effect of nationality on elective surgery by comparing Saudi and non-Saudi results. The findings among non-Saudis were not statistically significant, in contrast to the Saudis, whose findings regarding doctor recommendation and gender were statistically significant. Among Saudis, the influence of nationality on the gender of the physician was 2.39 times more likely to affect the choice of elective surgery. However, among Saudis, the influence of nationality on the recommendation was 2.14 times less likely to influence elective surgery. The influence of the gender of the doctor and the choice of elective surgery could be attributed to the Kingdom of Saudi Arabia's significant cultural heritage. Similar findings were reported in the Alotaibi et al. [2] and Yahanda et al. [8] studies.

Employment was the final demographic variable that exhibited a noteworthy impact in the study. Notably, the impact of employment was solely significant with respect to the gender of the physician. It was significant within the cohorts of those employed and those who were retired. The impact of employment status on the gender of the physician was found to have 2.32 times higher odds of affecting the decision to undergo elective surgery among those who were employed in comparison to those who were retired, who had 1.88 times greater odds of influencing the decision to undergo elective surgery. The study by Wakam et al. [3] reported findings that were similar. There was no statistically significant correlation between employment and the recommendation. The present study posits that the selection of elective surgery clinics in the Kingdom of Saudi Arabia may be linked to the stability of the employment sector and the post-retirement support provided by the government to pensioners. Consequently, the aforementioned cohorts possess the financial means to select their preferred clinics, in contrast to the underprivileged and jobless individuals within the community.

The study participants' socio-demographic data included gender and nationality. The disproportionate representation of the two socio-demographic groups may pose a challenge regarding the applicability of the results to the broader Saudi population. For example, the data indicates that 65% of the participants were female, whereas 35% were male. Similarly, with respect to nationality, 96% of the sample population were identified as Saudi nationals, while 5% were identified as non-Saudi nationals. The Kingdom of Saudi Arabia is frequently chosen as a destination by individuals hailing from Asia and Africa searching for improved economic prospects.

Consequently, it would be beneficial to record the perspectives of these cohorts regarding the pursuit of healthcare services, particularly with regard to elective surgical procedures and the selection of surgeons. Therefore, due to the lack of adequate data, it is impossible to generalize the present study's findings to non-Saudi populations. To enhance the comprehensiveness of future research, it is recommended to augment the sample size of male participants to facilitate comparative analysis with their female counterparts. Additionally, it is advisable to gather sufficient data on non-Saudi individuals, as this would help bridge the existing knowledge gap regarding selecting a surgeon for elective surgical procedures among foreign nationals.

## Conclusions

The current study aimed to explore factors that are the most common manners patients in the Kingdom of Saudi Arabia use to reach their appropriate surgeon. The gender of the doctor, age, gender of the patient, nationality, and employment are significant factors that would influence a patient's choice of preferred surgeon. The age and gender of the patient play a significant positive role in the relationship between the choice of the gender of the doctor and the selection of elective surgery in the Kingdom of Saudi Arabia. However, nationality and employment status play significant negative roles in the relationship between the choice of the gender of the doctor and the selection of elective surgery in the Kingdom of Saudi Arabia. In the Kingdom of Saudi Arabia, significant changes are observed in the choice of orthopedic and urology clinics, with the largest proportion inclining toward male patients compared to female patients.
